# Animal models for the study of depressive disorder

**DOI:** 10.1111/cns.13622

**Published:** 2021-03-01

**Authors:** Juhyun Song, Young‐Kook Kim

**Affiliations:** ^1^ Department of Anatomy Chonnam National University Medical School Hwasun Korea; ^2^ Department of Biochemistry Chonnam National University Medical School Hwasun Korea

**Keywords:** depression, depression animal model, depressive behavior, functional analysis, transcriptomic analysis

## Abstract

Depressive disorder is one of the most widespread forms of psychiatric pathology, worldwide. According to a report by the World Health Organization, the number of people with depression, globally, is increasing dramatically with each year. Previous studies have demonstrated that various factors, including genetics and environmental stress, contribute to the risk of depression. As such, it is crucial to develop a detailed understanding of the pathogenesis of depressive disorder and animal studies are essential for identifying the mechanisms and genetic disorders underlying depression. Recently, many researchers have reported on the pathology of depression via various models of depressive disorder. Given that different animal models of depression show differences in terms of patterns of depressive behavior and pathology, the comparison between depressive animal models is necessary for progress in the field of the depression study. However, the various animal models of depression have not been fully compared or evaluated until now. In this paper, we reviewed the pathophysiology of the depressive disorder and its current animal models with the analysis of their transcriptomic profiles. We provide insights for selecting different animal models for the study of depression.

## Graphical abstract

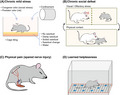

## 1 OVERVIEW OF DEPRESSIVE DISORDER

Depressive disorder is one of the major emerging psychiatric mood disorders, worldwide. It was reported that around 17% of people experience depression at least once in their lifetime.^1^ The symptoms and comorbidity of depression include social withdrawal, disturbed sleep, depressed mood (sadness), apathy, anxiety, changes in food consumption, psychomotor retardation, and memory deficits.^2^ Major depressive disorder is mainly characterized by consistently depressive mood, loss of pleasure, appetite pattern change, insomnia, behavior motor retardation, fatigue, and feelings of worthlessness for a minimum of 2‐week period.^3^

Depression is considered to be caused by the mutual influence of multiple psychological and social factors as well as epigenetic factors.^4^ Physical pain and chronic stress can induce depression and influence its progression and severity.^5^, ^6^ Cases of depression are heterogeneous in terms of genetic influences, clinical progression, neurobiological changes, and treatment responses to antidepressants.^7^, ^8^ In recent decades, studies of the progression of depression have reported on abnormalities in brain circuitry and on cellular and molecular alterations in the depressive brain.^9^, ^10^

**FIGURE 1:**
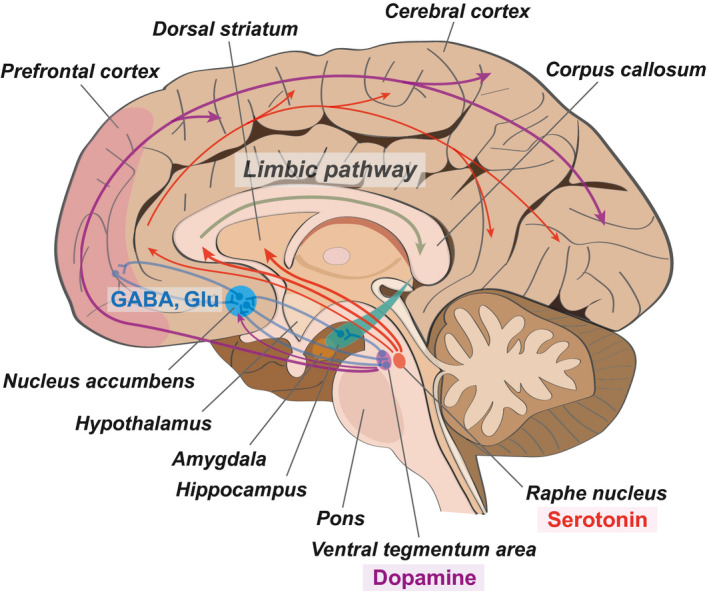
The neuroanatomical image of depression. This schematic image presents the important neurotransmitter pathway and the neuronal connection between different brain regions in depression. The nucleus accumbens plays as a critical connection hub in depression‐related brain regions. GABA (gamma‐aminobutyric acid) and Glu (glutamate), the neurotransmitters, contribute to the connective signal between the nucleus accumbens and the prefrontal cortex. Serotonin, which is secreted from the raphe nucleus of the brain stem, contributes to the limbic pathway and finally affects the hippocampus, related to cognitive function. Dopamine, another neurotransmitter, which is secreted from the ventral tegmentum area of the brain stem, influences the whole cerebral cortex region in the brain including the prefrontal cortex. See texts for the details. Red arrows indicate the serotonin pathway, and purple arrows indicate the dopamine pathway. Blue lines show the neuronal connection between different brain regions

Human neuroimaging studies and studies using animal models have reported that depression results from functional impairments in connections between various brain regions ^11^, ^12^ and that it is involved in the alteration of brain structures ^13^ (Figure 1). Such studies have also reported that depression is associated with alterations in the structure and functional morphology, as well as the modulation of cellular factors, such as transcription factors, in affected brain regions.^12^, ^14^ The nucleus accumbens is considered the main regulation center of neuronal circuits implicated in depression.^15^, ^16^ The nucleus accumbens integrates limbic and cortical information from the prefrontal cortex, ventral hippocampus, and the amygdalar region.^17^ Depression‐related brain regions also include the dorsal and medial prefrontal cortices, insular lobe, orbitofrontal cortex, amygdala, hippocampus, and cingulate cortex.^18^, ^19^, ^20^ Decreases in metabolism, in these brain regions, promote the onset of depression and greatly accelerate its progression.^21^, ^22^ Previous studies have reported that decreases in metabolism, accompanied by reductions in blood flow, are positively correlated with reduced brain volume in these brain regions and with depression.^18^, ^19^, ^23^, ^24^, ^25^, ^26^

Dopaminergic neuron in the ventral tegmental area mainly produces dopamine and projects to other brain areas including the striatum and mesolimbic reward pathways related to pain relief center (Figure 1). Dopamine neurons in the ventral tegmental area are key to mediate stress response.^27^ Also, dopamine neuron in pars compacta of substantia nigra contributes to cortico‐basal ganglia‐thalamocortical circle.^27^, ^28^ Generally, pain contributes to various brain regions, such as the prefrontal cortex, anterior cingulate cortex, dorsal and ventral striatum, and amygdala.^29^ Dopamine projected to nucleus accumbens could block physical somatosensory pain.^30^

Serotonin neuron from raphe nucleus projects to thalamus, limbic pathway, and prefrontal cortical regions and affects depressive‐like behaviors such as anxiety (Figure 1).^31^ Serotonin influences the activity of food eating, motor function, and anxiety feeling.^32^

Nucleus accumbens has glutamatergic neurons and GABAergic neurons, and is linked to depressive behavior‐related brain regions such as the ventral tegmental area, prefrontal cortex, and striatum (Figure 1).^33^, ^34^ Glutamatergic neurons in the nucleus accumbens project to the prefrontal cortex, hippocampus, amygdala, and basal ganglia, and influence mood and anxiety feeling.^35^ GABAergic neurons in the nucleus accumbens project to the prefrontal cortex, thalamus, hippocampus, and amygdala and affect the mesocorticolimbic reward system. These neurons regulate dopamine release and reward system, and contribute to memory function by activating the hippocampus and prefrontal cortex.^36^, ^37^

Based on these findings, many researchers have endeavored to identify meaningful biomarkers for depression, in order to diagnose depression and identify its stage of progression. Suggested biomarkers include peripheral blood‐based biomarkers,^38^ pro‐inflammatory factors,^39^, ^40^, ^41^ neurotrophic factors,^42^, ^43^ vascular endothelial growth factor,^44^ neurotransmitters,^45^ lipid profile,^46^ and hypothalamus pituitary adrenal (HPA) axis biomarkers, including cortisol.^47^ In particular, epigenetic markers may be key to characterize the pathology of depression, because epigenetic alterations lead to change in the production of proteins related to depressive behavior.^48^ Some studies have demonstrated that the accelerated shortening of telomeres is related to stress,^49^, ^50^ anxiety,^51^ and depressive‐like behavior.^52^ Other researchers have emphasized the strong contribution of genetic factors to the pathogenesis of depression and individuals’ susceptibility to it.^53^ However, it has not been possible to identify predictors of any value at the onset of depression or during its later stages, until now. This is because depression is a biologically heterogeneous psychiatric disease that is associated with a diverse array of potential causes and symptoms. These include anxiety, chronic stress, traumatic experiences, neurochemical reconfigurations, and genetic susceptibility.

One study has also reported that the brains of depressed individuals are commonly under oxidative stress resulting from the overproduction of reactive oxygen species. Further, damage to proteins, lipids, and cell DNA was observed, as was cell death.^54^ Other studies have suggested changes in growth factors and inflammatory proteins and stress‐related enzymes and protein interaction system in the depressive brain.^55^, ^56^ By combining knowledge from these and other studies, it may be possible to identify biomarkers that can be used to make specific, reliable predictions regarding the course of depression. In turn, these predictions could shape the development of suitable, individualized treatments for patients with depression. The detection of early changes in the brains of patients with depression could allow us to better manage the progression of depressive symptoms.

## 2 DIVERSE ANIMAL MODELS FOR THE STUDY OF DEPRESSION

In order to study the neural mechanisms underlying depression, researchers have developed various animal models for depression. These include a model emphasizing unpredictable chronic stress exposure,^57^ the learned helplessness model,^58^ a model emphasizing the role of depression induction via the exogenous administration of glucocorticoids,^59^ the olfactory bulbectomy depression model,^60^ the social defeat model,^61^ and a model emphasizing genetic manipulation.^9^, ^62^, ^63^ These models have shown depressive‐like behavior similar to that in human patients with depression. However, each animal model of depression has advantages and disadvantages and accounts for slightly different depression symptoms.^64^ Thus, a comprehensive review and analysis of each animal model would be helpful for future research. We outlined the differences between these depression models which may help other researchers to choose a suitable animal model for the study of depression (Table 1).

**TABLE 1:** Differences between the depression models.

	Inducing factors	Applicable human symptom
Chronic mild stress	Congener odor, predator odor, cage tiling, sawdust change, confinement	Sleep disturbance, depressive‐like behavior, reward response, anhedonia
Chronic social defeat	Visual stress, olfactory stress, physical contract	Reduction of locomotor activity, reduction of enthusiastic behavior, anxiety, submissive behavior, social avoidance, reduction of food eating
Physical pain	Physical pain (spared nerve injury)	Neuropathic and nociceptive pain
Learned helplessness	Electronic stress, continuous involuntary movement	Symptoms of traumatic stress, comorbid major depression

**FIGURE 2:**
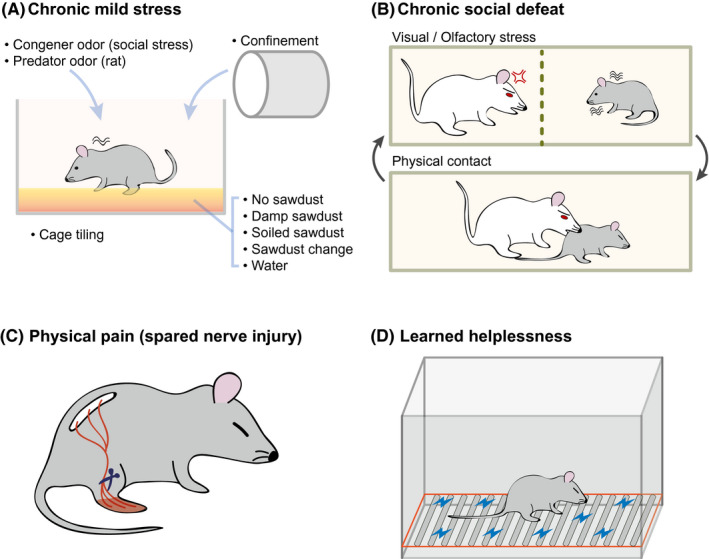
Experimental models for the study of depression. (A) Chronic mild stress. In this model, the mice are exposed to a series of low‐intensity stressors at unpredictable times for 9 weeks. (B) Chronic social stress. In this model, depression is induced over 10 days by directly exposing the experimental mouse to a larger and aggressive mouse for 5 minutes a day and then housing across a transparent barrier to sustain sensory contact. (C) Physical pain. A spared nerve injury is surgically inflicted, resulting in depressive behaviors due to persistent neuropathic pain. (D) Learned helplessness. The mouse is exposed to unpredictable and inescapable electric footshocks for two consecutive days, after which the mouse shows a defect in its escape behavior and depressive symptoms

Previous studies have reported on the neuropathology of depression based on various animal models (Figure 2). There are public resources that analyzed the transcriptomic profile for each animal model of depression. For the comparison of transcriptome among diverse depression models and provide easy access to these data, we obtained the RNA sequencing data and calculated gene expression levels (Supplementary Figure 1).^65^, ^66^, ^67^, ^68^ These data include the profiling of changes in gene expression in several brain regions, including the prefrontal cortex, hypothalamus, and nucleus accumbens, which are related to the progression of depression, as described above (Table [Link], [Link], [Link], [Link], [Link].) For each depression model, we reviewed the pathophysiology of each model and presented the functional changes based on gene expression for these brain regions as described below.

### 2.1 Chronic mild stress model

Chronic mild stress is the most common cause of depressive mood disorders. It results in multiple physiological changes in the brain, including the alteration of corticosterone regulation through the HPA axis, impaired neurogenesis, synaptic dysfunction, and gene expression changes.^69^, ^70^, ^71^ It also induces behavioral changes including depressive‐like behavior, a reduction in the reward response, and sleep disturbances.^72^, ^73^ Several studies showed that animals exposed to repetitive stress display behavioral changes in open field behavior tests ^57^ and a decrease in saccharin or sucrose fluid consumption, which is considered indicative of anhedonia.^74^, ^75^ It was also shown that chronic, uncontrollable stress contributes to the impairment of the brain stimulation reward system.^76^ Therefore, these reports suggest that the chronic stress animal model can be used to study depressive neuropathology,^77^ and several studies used this model for the test of therapeutic targets to treat the depressive disorder.^78^, ^79^

**FIGURE 3:**
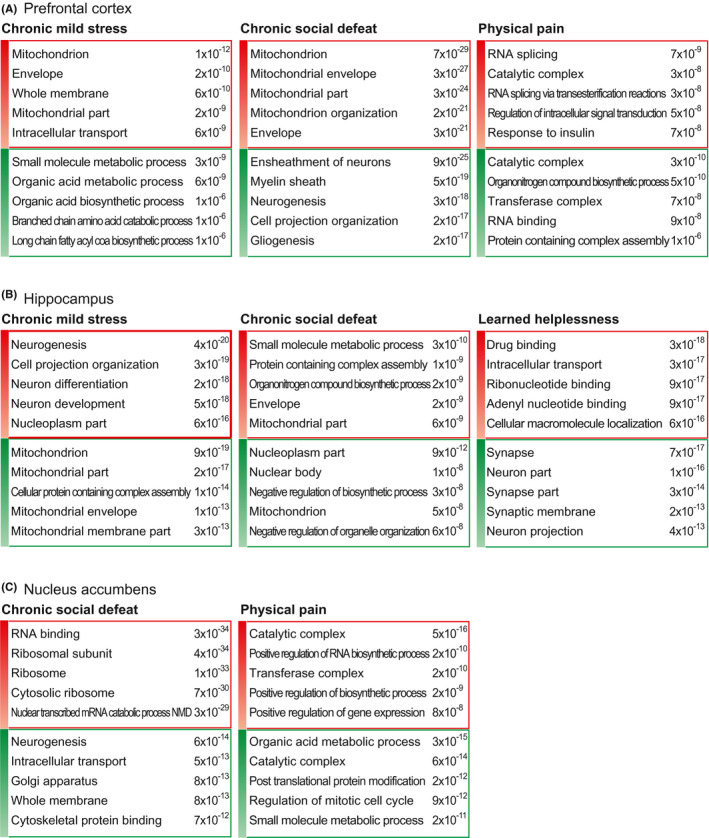
Gene ontology analysis of differentially expressed gene groups for each model. First, we selected the top 10000 genes for each model, based on their average signal (FPKM) from RNA sequencing data. If there was any sample with an FPKM value of zero, we removed the genes from further analysis. We selected the top 200 increased (red color box) and decreased genes (green color box) based on their fold changes between the depression model mouse and its corresponding control mouse. We then used these gene groups for gene ontology (GO) analysis with MSigDB (http://software.broadinstitute.org/gsea/msigdb/). Based on the p‐value, we selected the top 20 GO terms (Supplementary Table 6). We present the most significant five terms in this figure

In the mouse model of chronic mild stress, the mice are subjected to unpredictable mild psychosocial stressors for 9 weeks (Figure 2A).^80^ From the transcriptome data, many genes showed marked expression changes in brain region including the prefrontal cortex and hippocampus, in chronic mild stress animal models (Table S2).^65^ Interestingly, the gene ontology (GO) terms related to mitochondria and membranes were enriched in the increased genes group, for the prefrontal cortex, but the same terms were identified in the decreased genes group, for the hippocampus (Figure 3A,B, and Table S6). Related to this result, many studies have shown the connection between mitochondria and depression.^81^, ^82^ Moreover, the GO terms related to neurogenesis were enriched in the increased genes group, for the hippocampus. Neurogenesis may be altered in animals with chronic stress, as suggested above.^71^

### 2.2 Chronic social defeat model

The chronic social defeat animal model has been used to study the pathology of depression and its underlying mechanisms.^83^, ^84^ The chronic social defeat model is characterized by decreases in locomotor activity,^85^ reductions in enthusiastic and aggressive behavior,^86^ and increases in submissive behavior and anxiety,^87^ as is observed in humans with depression. These symptoms ultimately lead to an increased risk of depression progression.^61^ Furthermore, morphologically, the chronic social defeat model featured a reduction in neuronal cell proliferation and a decrease in hippocampus volume.^88^, ^89^ It was also demonstrated that the chronic social defeat model could alter reward circuity and cause changes in the brain, associated with increased susceptibility to engaging in depressive behavior.^90^ Moreover, the chronic social defeat model altered the activity of dopaminergic neurons in the ventral tegmental area and ultimately resulted in social avoidance and a reduced preference for sucrose, as is expected in depression pathology.^33^, ^91^, ^92^ Other studies also showed that chronic social defeat stress leads to functional and structural changes in neural circuitry.^12^, ^84^ In particular, it was demonstrated that the ventral hippocampus and nucleus accumbens were more susceptible to stress from chronic social defeat than was the prefrontal cortex.^66^

In the previous work, it was established that chronic social defeat stress induces susceptible and resilient phenotypes in a ratio of 2 to 1, respectively.^84^ The susceptible phenotype showed enduring social avoidance, and the resilient phenotype exhibited a tendency of social interaction similar to control mice. Therefore, the expression data of susceptible phenotype were only used for the following analysis. Based on the transcriptome of the prefrontal cortex, hippocampus, nucleus accumbens in the chronic social defeat model^66^ (Table S3), the result of GO analysis for each gene group is presented (Figure 3 and Table S6). In the increased genes group, the GO terms related to mitochondria were enriched for the prefrontal cortex (Figure 3A). Moreover, the mitochondrion term was also detected in the decreased genes group for the hippocampus. These results are quite similar to those observed in the chronic mild stress model. However, the terms related to neurogenesis and the myelin sheath were enriched in the decreased genes group for the prefrontal cortex in the chronic social defeat model. Some of these terms were identified in the increased genes group for the hippocampus in the chronic mild stress model (Figure 3A,B). Further, the small molecule metabolic process term was the most highly enriched in the increased genes group for the hippocampus in the chronic social defeat model. The same term was the most highly enriched in the decreased genes group for the prefrontal cortex in the chronic mild stress model. We suggest that chronic mild stress and chronic social defeat models have both common and opposite molecular alterations in the prefrontal cortex and hippocampus, respectively. For the nucleus accumbens, in the chronic social defeat model, the terms related to RNA and ribosomes were included in the increased genes group while those related to neurogenesis and membranes were included in the decreased genes group (Figure 3C). Because the neurogenesis was decreased both in the prefrontal cortex and the nucleus accumbens in this model, it is reasonable to expect that a similar molecular change, related to decreased neurogenesis, occurs in these areas.

### 2.3 Physical pain model

Physical pain is another major cause of depression. Neuropathic and nociceptive pain, especially, increase the risk of developing depression.^93^, ^94^ Approximately one‐fifth of the general population currently suffers from chronic pain.^95^ Based on these epidemiological data, we assume that many people are likely to have depressive symptoms due to physical pain. Specifically, pain caused by damage to sensory nerve pathways has been shown to influence depressive moods and to be involved in neuronal cell death at brain regions linked to depression, including the insular lobe, prefrontal cortex, thalamus, hippocampus, anterior cingulate, and amygdala.^96^

It was reported that the prefrontal cortex and nucleus accumbens experience neuronal cell death during pain, which subsequently led to the development of depression.^97^ The nucleus accumbens is connected to several brain regions related to depressive‐like behavior and pain regulation, including the ventral tegmental area, thalamus, prefrontal cortex, and amygdala ^33^, ^34^ (Figure 1). Several neuroimaging studies have demonstrated that patients suffering from chronic physical pain differed from healthy individuals in terms of activity in the nucleus accumbens and prefrontal cortex, which play a role in reward processes.^98^, ^99^, ^100^ Synaptic dysfunction was also reported in the prefrontal cortex, due to neuropathic pain, in the relevant animal model.^34^, ^101^, ^102^ It has also been found that chronic physical pain is strongly associated with areas ranging from the ventromedial prefrontal cortex to the periaqueductal gray. This region represents the control center for descending sensory pain modulation and has pain‐reducing enkephalin‐producing cells in humans ^103^ and rodents.^104^ Thus, chronic pain and depression show common changes in neuroplasticity mechanisms and are strongly linked to each other. The physical pain model can provide more understandable information toward developing treatments for depression.

The GO analysis using the transcriptome of the prefrontal cortex and nucleus accumbens in the chronic pain mouse model 2.5 months after the injury was performed ^67^ (Figure 3A,C, and Tables [Link], [Link]). Interestingly, the GO term “catalytic complex” was enriched both in the increased and decreased genes groups for the prefrontal cortex and nucleus accumbens. However, there was no notable overlap of GO terms between the physical pain model and other models. This maybe is because the physical pain model is a model of depression induced by physical surgery, whereas psychological stimulation is induced in the other depression models (Figure 2).

### 2.4 Learned helplessness model

The learned helplessness model has been used to make predictions in cases of depression because it accounts for the symptoms of traumatic stress disorder and comorbid major depression.^105^, ^106^ Learned helplessness features symptoms of depression that affect neurochemical and molecular processes. These include increased inflammation and the cell death of norepinephrine neurons in the locus coeruleus region, leading to depressive behavioral consequences.^107^

In the learned helplessness mice model, 360 scrambled electric footshocks (0.15 mA) with varying duration (1–3 seconds) and interval (1–15 seconds) are treated for two consecutive days.^108^ From the GO analysis of the transcriptome from the learned helplessness model,^68^ the most enriched terms in the decreased genes group, for the hippocampus, included those related to the synapse (Figure 3B, and Table [Link], [Link]). A previous study reported on the remodeling of synapses in the learned helplessness depression model.^109^ However, no GO terms that were enriched in the learned helplessness model were identified in the GO analysis of the hippocampus in other depression models (Figure 3B). We expect that this depression model is characterized by different molecular changes in the hippocampus, compared with other depression models including the chronic mild stress and chronic social defeat models.

Based on the description above, it is obvious that there are commonalities and differences across the many animal models of depression, in terms of changes in gene expression profiles and depressive pathology, in each brain region. One of the notable conclusions from the GO analysis is that the difference in gene expression among depression models is greater than that among tissues that we analyzed such as the prefrontal cortex, hippocampus, and nucleus accumbens (Figure S2). Thus, although there were many functional terms commonly affected across the different models, our findings indicate that we should consider the difference among the depression animal models, and that it is important to choose a proper model to study depressive disorder. We found that chronic mild stress and chronic social defeat models show very similar molecular changes for the gene group with the greatest changes in gene expression. In contrast, the physical pain model had no specific terms in common with the other depression models. This suggests that the selection of a suitable model is required based on the type of depressive disorder that the researcher wants to study.

## 3 COMMONLY CHANGED GENES AMONG DIFFERENT DEPRESSION MODELS

There were considerable differences, in terms of changes in gene expression, among the depression models. To offer a list of commonly altered genes among these models, we cross‐compared the gene groups which were significantly altered for each model (Figure S3 and Table S7). Among the models compared, the chronic mild stress and chronic social stress models had the most genes in common, as was expected based on the common GO terms shared between these two depression models (Figure 3 and Figure S3). Heat shock protein family B (small) member 11 (Hspb11) was the only gene commonly decreased in the prefrontal cortex across three depression models (Figure S3A). Interestingly, the HSPB11 locus was reported as one of the most highly hypermethylated regions associated with major depressive disorder.^110^ Because protein misfolding and aggregation are observed in a diverse array of neuronal disorders, we expect that Hspb11 also plays a role in the pathology of depression.^111^

Among some of the other genes commonly detected across two depression models, there were previous reports which showed the roles of those genes in depression. Neuronal PAS domain protein 4 (Npas4), whose expression was decreased in the prefrontal cortex in the chronic social stress and physical pain models, was reported to play a critical role in depressive behavior, in a study using knockout mice (Figure S3A).^112^ A recent co‐expression analysis suggested that ATP5G1 is associated with major depressive disorder,^113^ and that a functional polymorphism in the promoter of XBP1 was reported to be associated with depressive episodes ^114^ (Figure S3B). It was also shown that hippocampal SPARC, which was included as an increased gene in the prefrontal cortex, regulated depression‐related behavior ^115^ (Figure S3B). Among the other genes, there was a report that the G protein regulated inducer of neurite outgrowth 1 (Gprin1) gene is involved in neurite outgrowth ^116^ and that YTH domain family 2 (Ythdf2) is linked to the control of neuronal development.^117^ However, the relationship of these genes with depression is still unknown (Figure S3C,F). Interestingly, timeless interacting protein (Tipin) was identified as a commonly decreased gene in the prefrontal cortex and nucleus accumbens, in both the chronic social stress model and the physical pain model.

As described above, several genes were commonly altered across the different depression models. However, most differentially expressed genes were included in one group exclusively. Therefore, in addition to the study of common genes, the elucidation of the function of these genes is necessary to identify the differences between the depression models. We also note that the change of gene expression in different brain regions is not enough. Since there may be differences in gene expression patterns for each cell type, it is necessary to analyze the difference in expression patterns between cell types (stimulatory, inhibitory, or modulatory neurons) in each brain region. This process will be helpful to get a comprehensive understanding of the pathology of depressive disorder.

## 4 CONCLUSION

The review of previous studies and the additional analysis presented above show the commonalities and differences between depression models that have widely been used in studies of depressive disorder. These models are influenced by different environmental and physical factors and show slightly different neuropsychiatric features. However, we observed that these diverse depression animal models share altered genes related to the aggravation of neuroinflammation and synaptic dysfunction. On the other hand, it also should be noted that changes in gene expression are generally quite different across the depression models. Consequently, we suggest that researchers must consider the differences between the models when deciding which depression model best fits their purposes. Our analysis could contribute to informing such decisions. Further, a more in‐depth research is necessary to identify the animal model of depression that is most comparable to human patients with depression.

## CONFLICT INTEREST

The authors declare that they have no competing interests.

## ETHICS APPROVAL AND CONSENT TO PARTICIPATE

Not applicable.

## AUTHOR CONTRIBUTIONS

Y‐K. K.: methodology. J. S.: formal analysis. J. S. and Y‐K. K.: conceptualization, investigation, writing—original draft preparation, writing—review and editing, and funding acquisition. All authors have read and agreed to the published version of the manuscript.

## CONSENT FOR PUBLICATION

Not applicable.

## Supporting information

Table S1

Table S2

Table S3

Table S4

Table S5

Table S6

Table S7

Supplementary Material

## Data Availability

The data that support the findings of this study are available in the supplementary material of this article.
