# Supplementary material for: Animal models for the study of depressive disorder
Source: CNS Neurosci Ther. 2021 Mar 1;27(6):633–42. doi: 10.1111/cns.13622 (PMC8111503; doi:10.1111/cns.13622)
Supplement: Supplementary file 8 — Supplementary Material [file CNS-27-633-s008.docx]

**Supplementary Information**

**Animal models for the study of depressive disorder**

Juhyun Song, Young-Kook Kim

**Supplementary Tables**

Table S1. Statistical analyses of RNA sequencing libraries.

Table S2. Changes in gene expression for the chronic mild stress model.

Table S3. Changes in gene expression for the chronic social stress model.

Table S4. Changes in gene expression for the physical pain model.

Table S5. Changes in gene expression for the learned helplessness model.

Table S6. The top 20 GO terms for differentially expressed genes for each depression model.

Table S7. The intersection of commonly altered genes among different depression models.


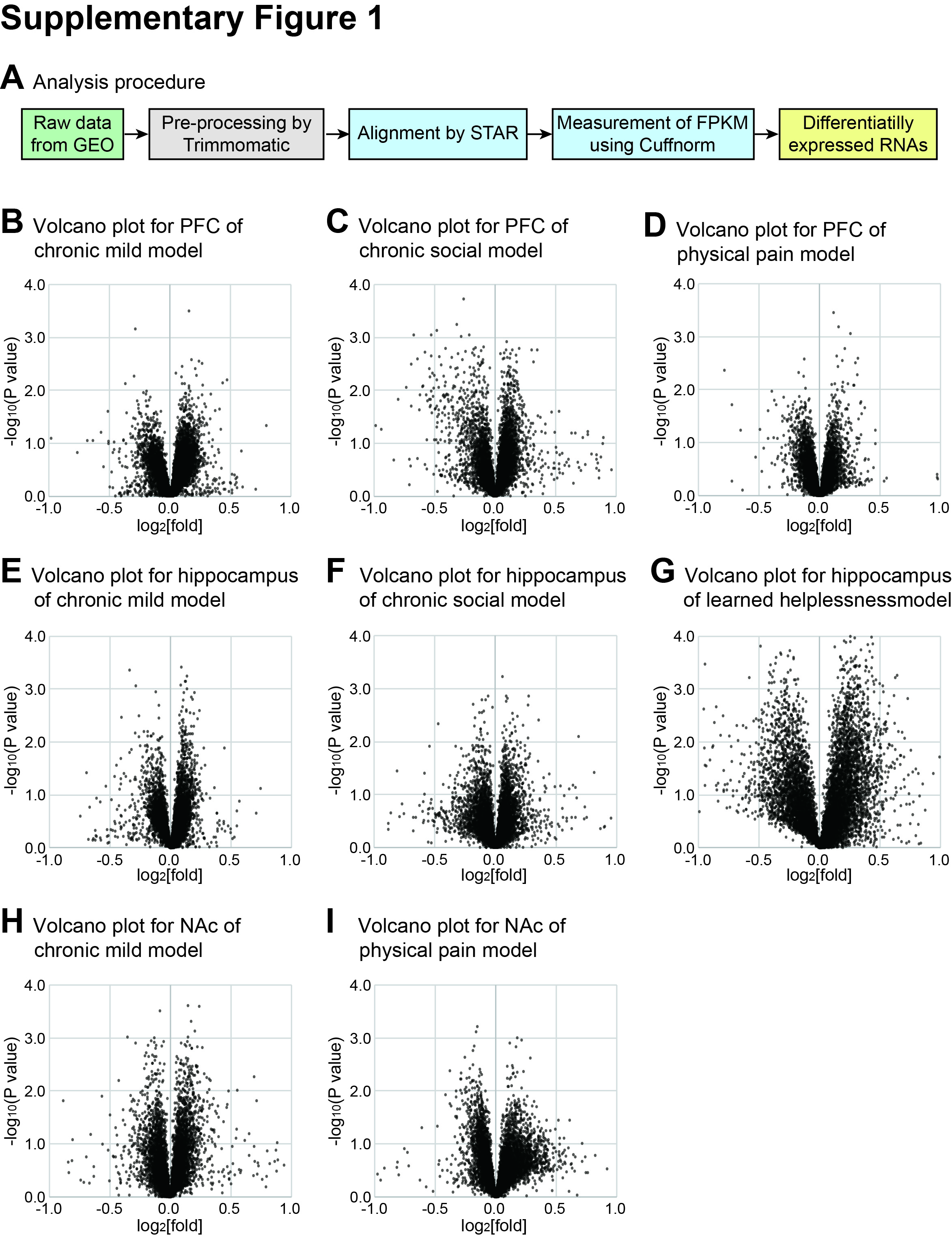


**Supplementary Figure S1.** (A) RNA sequencing analysis procedures. The raw data for each RNA sequencing analysis were downloaded from Gene Expression Omnibus (GEO) and the low-quality reads were discarded using the Trimmomatic algorithm (www.usadellab.org/cms/?page=trimmomatic). The sequencing reads were aligned to the mouse genome by STAR (github.com/alexdobin/STAR) and fragments per kilobase of transcript per million mapped reads (FPKM) were calculated by Cuffnorm (cole-trapnell-lab.github.io/cufflinks). (B-I) The volcano plots for illustrating the distribution of gene expression changes in each dataset.


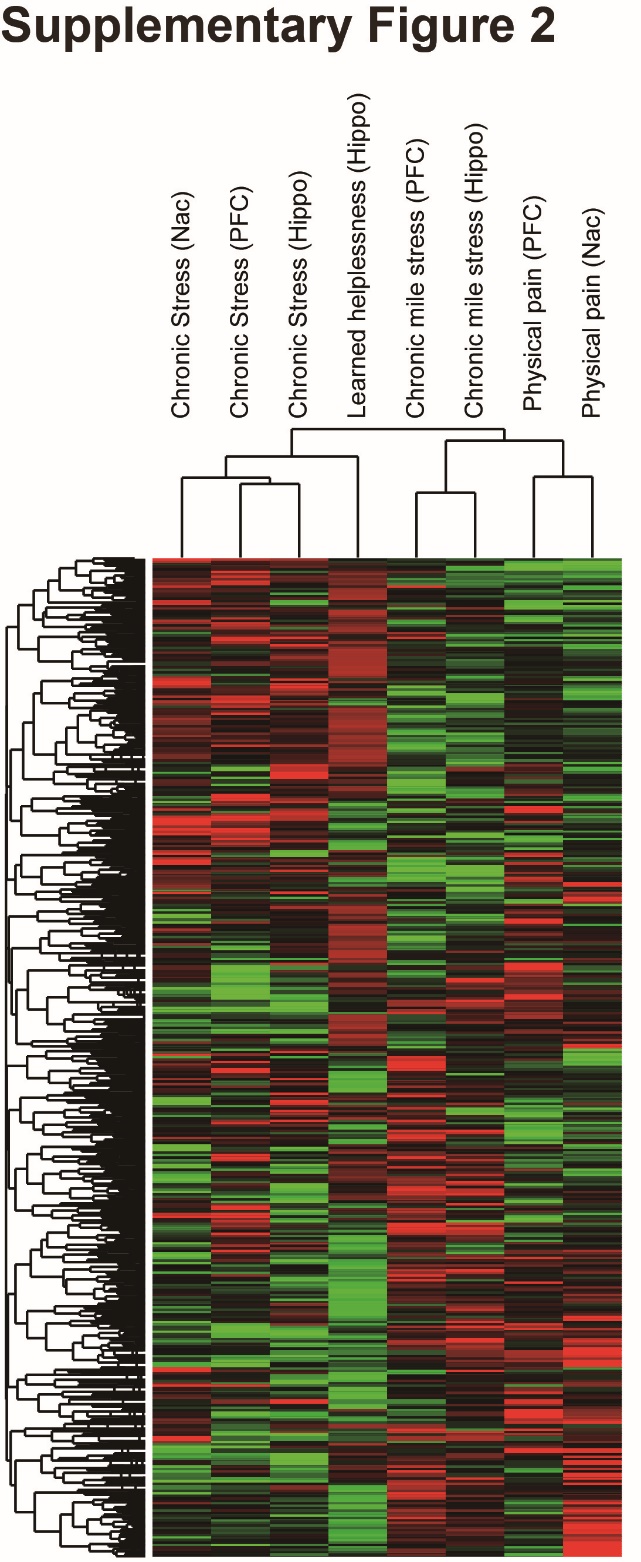


**Supplementary Figure S2.** Heat map depicting the result of the hierarchical clustering analysis. The log value of fold changes in gene expression from Supplementary Tables 2 to 5 were combined. Cluster 3.0 (bonsai.hgc.jp/~mdehoon/software/cluster/) was used to perform hierarchical clustering with the complete linkage method and with the similarity matrix of centered correlation. The result was depicted using Java TreeView (jtreeview.sourceforge.net).


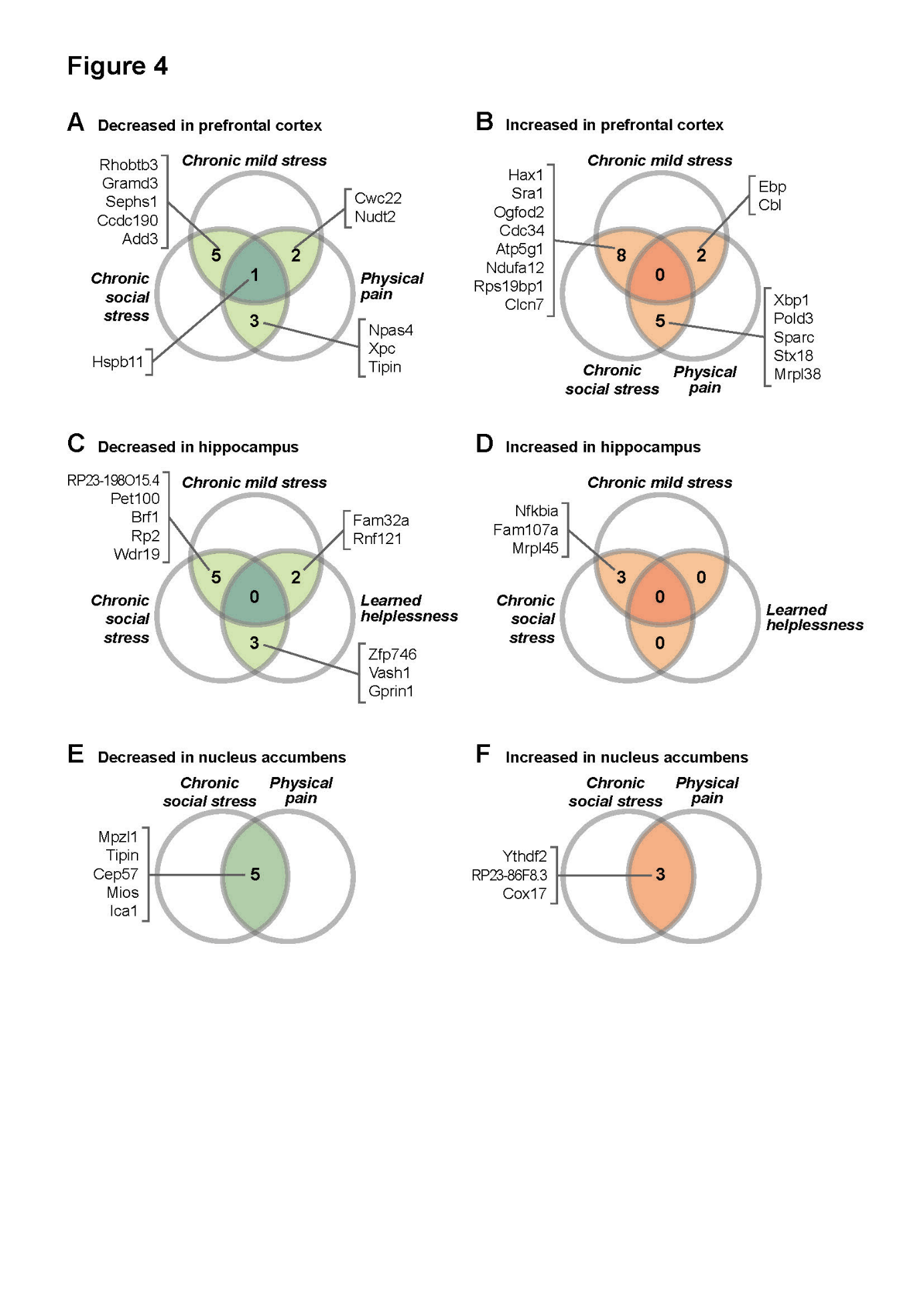


**Supplementary Figure S3. Commonly changed genes among depression models in each brain region related to depression.** In the prefrontal cortex, the most commonly (A) decreased or (B) increased genes among three depression models are shown. Similar gene groups are shown for (C) decreased or (D) increased genes in the hippocampus, and (E) decreased or (F) increased genes in the nucleus accumbens. We selected commonly altered genes among the top 200 increased and decreased genes (Figure 3). The changes in gene expression regarding the genes in this figure are described in Supplementary Table 7.
